# Per1 gene polymorphisms influence the relationship between brain white matter microstructure and depression risk

**DOI:** 10.3389/fpsyt.2022.1022442

**Published:** 2022-11-11

**Authors:** Rui Zhao, Jin-Bo Sun, Hui Deng, Chen Cheng, Xue Li, Fu-Min Wang, Zhao-Yang He, Meng-Ying Chang, Li-Ming Lu, Chun-Zhi Tang, Neng-Gui Xu, Xue-Juan Yang, Wei Qin

**Affiliations:** ^1^School of Electronics and Information, Xi’an Polytechnic University, Xi’an, China; ^2^Intelligent Non-Invasive Neuromodulation Technology and Transformation Joint Laboratory, Xidian University, Xi’an, Shaanxi, China; ^3^Engineering Research Center of Molecular and Neuro Imaging of the Ministry of Education, School of Life Sciences and Technology, Xidian University, Xi’an, Shaanxi, China; ^4^Guangzhou Institute of Technology, Xidian University, Xi’an, Shaanxi, China; ^5^South China Research Center for Acupuncture and Moxibustion, Medical College of Acu-Moxi and Rehabilitation, Guangzhou University of Chinese Medicine, Guangzhou, China

**Keywords:** PER1 gene polymorphisms, diffusion tensor imaging, Beck Depression Inventory, depressive risk, tract-based spatial statistics

## Abstract

**Background:**

Circadian rhythm was involved in the pathogenesis of depression. The detection of circadian genes and white matter (WM) integrity achieved increasing focus for early prediction and diagnosis of major depressive disorder (MDD). This study aimed to explore the effects of PER1 gene polymorphisms (rs7221412), one of the key circadian genes, on the association between depressive level and WM microstructural integrity.

**Materials and methods:**

Diffusion tensor imaging scanning and depression assessment (Beck Depression Inventory, BDI) were performed in 77 healthy college students. Participants also underwent PER1 polymorphism detection and were divided into the AG group and AA group. The effects of PER1 genotypes on the association between the WM characteristics and BDI were analyzed using tract-based spatial statistics method.

**Results:**

Compared with homozygous form of PER1 gene (AA), more individuals with risk allele G of PER1 gene (AG) were in depression state with BDI cutoff of 14 (*χ^2^* = 7.37, uncorrected *p* = 0.007). At the level of brain imaging, the WM integrity in corpus callosum, internal capsule, corona radiata and fornix was poorer in AG group compared with AA group. Furthermore, significant interaction effects of genotype × BDI on WM characteristics were observed in several emotion-related WM tracts. To be specific, the significant relationships between BDI and WM characteristics in corpus callosum, internal capsule, corona radiata, fornix, external capsule and sagittal stratum were only found in AG group, but not in AA group.

**Conclusion:**

Our findings suggested that the PER1 genotypes and emotion-related WM microstructure may provide more effective measures of depression risk at an early phase.

## Introduction

Major depressive disorder (MDD) is one of the most common psychiatric disorders (1–3). According to the World Health Organization, MDD ranks among the top causes of worldwide disease burden and disability (4, 5), with an estimated lifetime prevalence of 20.6% in the general population (6) and with the peak ages of diagnosis from mid-adolescence to mid-40s (7, 8). To reduce that burden, earlier diagnosis, and interventions, as well as identification of those young people who are at high risk of MDD has been prioritized (9–11).

Recently, many lines of evidence in humans or animal models clearly demonstrated a close relationship between MDD and the disturbances of sleep-wake and circadian systems (12–17). The hypothesis that dysregulations of circadian rhythm may play a critical role in the pathophysiology of MDD, is supported by recent findings in the field of molecular biology and genetics of the complex machinery regulating biological clocks (13, 18–21). Therefore, disturbed circadian function have been suggested to be a major risk factor in the development of MDD (14, 18, 22–25). Focused on the people who are vulnerable to the disturbances of circadian rhythm will provide pivotal evidence in the prevention of MDD.

At the gene level, accumulated evidence have implicated that altered circadian gene expression might represent a vulnerability factor for depression (19, 26–28). Based on the chronic mild stress (CMS) animal model of depression, previous studies have indicated that the diurnal oscillation of the expression of several circadian rhythm genes (such as CLOCK, CRY2, PER1, and PER3, etc.) were disturbed and its degree directly was correlated with mood-related behavior (29–31). However, the animal model of depression did not model the full syndrome of depression. Several studies have explored the relationship between depression and circadian rhythm genes in human. Previous studies have found disruptions of the rhythmic expression of PER1, PER2, CRY1, BMAL1, NPAS2, and GSK-3b in MDD, which might influence the susceptibility to recurrence after antidepressant treatment (32). Moreover, a study of postmortem human brains have reported that daily rhythmic patterns of circadian gene expression in the brain were seriously disrupted and/or desynchronized in MDD subjects, further confirming a key connection between circadian gene variation and MDD (19). On the other hand, a number of studies have tested links between circadian gene polymorphisms and the susceptibility to MDD, and several specific single-nucleotide polymorphisms (SNPs) were involved (33, 34). These studies suggested that clock gene alleles, which caused a modification of the circadian clock mechanism, might directly contribute to the onset of mood disorders, or might elicit a misalignment/disruption of the circadian system under the detrimental environment (35).

Due to the genetic architecture of depression is complex and genetic effects are not always saliently expressed at a behavioral level (36), the integration of genomics and neuroimaging techniques (imaging genetics) on MDD might help to identify biomarkers of genetic susceptibility and highlight brain changes mediated by underlying genetic factors (37–41). Recently, using diffusion tensor imaging (DTI) technique and tract-based spatial statistics (TBSS) method, many researches have focused on the interaction effect between risk genes and abnormalities of white matter (WM) integrity or fiber tracts in MDD, such as 5-HTTLPR heterozygotes (42, 43), BDNF-met carriers (44), SLC6A15-A carriers (45), NTRK2 homozygous A (46), FKBP5-T carriers (47), COMT homozygous G (48, 49), VMAT1 Thr136Ile-A carriers (50), THP2 homozygous G (51), RERE homozygous T (52). In addition, significant correlations have been observed between the MDD polygene risk score and fractional anisotropy (FA) values, as well as between SLC6A4, COMT or BDNF promoter region DNA methylation and WM microstructure (53–56). Furthermore, in terms of circadian gene, Bollettini et al. have found that CLOCK C carriers showed widespread increased mean diffusivity (MD) in several WM tracts compared to T homozygotes, and that PER3^4/4^ homozygotes showed significant increased radial diffusivity (RD) and reduced FA in several brain WM tracts compared with PER3^5/5^ homozygotes in depressed bipolar patients, but no significant interaction effect of the two clock genes on WM microstructure (57). Among the clock genes involved in the control system of circadian rhythms, PER1 gene aroused our interest, because its rhythmic expression was disrupted in MDD subjects (19, 30, 32), as well as polymorphisms of rs7221412 was associated with the timing of human behavioral rhythms (mean activity timing was delayed by 67 min in rs7221412*^GG^* versus rs7221412*^AA^* homozygotes) and time of death (58). However, to our knowledge, the interaction effects between PER1 gene polymorphisms and the WM microstructural abnormalities in MDD patients or susceptible population have not been depicted.

Considering that the dysregulated expression of circadian genes in MDD are mainly selectively altered in emotion-related brain regions (19), we aimed to explore the interaction effects of polymorphisms of PER1 gene (rs7221412) and depression level on the WM microstructural integrity in a group of university students. We hypothesize that the PER1 gene polymorphisms will modulate the relationship between depression level and WM microstructural integrity in emotion-related brain regions.

## Materials and methods

### Participants

100 healthy college students (all right-handed) were originally recruited from Xidian University in China via advertisements. The subjects would be excluded if they met the following conditions: (1) incomplete information or data (nine subjects failed to acquire the genotype and five subjects did not collect image data); (2) current or chronic illness (excluded four subjects); (3) traces of drugs (including excessive alcohol, nicotine, and caffeine use); (4) personal or family history of neurological or psychiatric disease (3 participants were removed); and (5) sleep disorders (such as sleep apnea, and nocturnal myoclonus, 2 subjects were discarded). At last, 77 subjects (50 men and 27 women; mean age: 20.94 ± 2.09 years, range from 18 to 27 years) were included in this study. Recruitment and screening took place between August 2018 and June 2019 and all data collection took place between March 2019 and January 2020. Each subject was required to finish the venous blood collection, behavioral questionnaires and Magnetic Resonance Imaging (MRI) scanning. In order to make sure subjects are in the same state during the experiment, we performed the behavioral data collection and MRI scanning within a day, and administered the venous blood collection in 3 days. The study protocol and all experimental procedures were reviewed and approved by the Institutional Research Ethics Committee of the Xijing Hospital of the Fourth Military Medical University for research on human subjects and performed in accordance with the Declaration of Helsinki. Written informed consent was obtained from all subjects prior to the study.

### PER1 polymorphism identification and genotyping

Two milliliter venous blood was prepared for genomic DNA extraction by using Blood Genomic DNA Kit (TIANGEN BIOTECH CO., LTD., Beijing, China) according to the manufacturer’s instructions. Polymorphism in period homolog 1 (*PER1*) gene were sequenced. Primers used for the polymerase chain reaction were as follows. *PER1* (rs7221412): 5’-ATGTGGGCAATAATACATAAGCA-3’ (forward), 5’-CTATTGACCATTACTTCGTGGA-3’ (reverse). The gene sequencing was performed in the laboratories of Nanjing JINSIRUI Bio-tech Co., Ltd. (Nanjing, China). In our study cohort, the distribution of polymorphism was consistent with the Hardy-Weinberg Equilibrium (χ^2^ = 0.941, *P* = 0.332).

### Behavioral data acquisition

The Beck Depression Inventory (BDI) (59) has been employed to measure the depressive level (60–64). Two depressive states were identified with the most commonly used cutoff BDI = 14 (BDI < 14, without depression; BDI ≥ 14, with depression) (65). The Munich ChronoType Questionnaire (MCTQ) were employed to quantify the phenotype of circadian rhythm (66). In this study, four index of MCTQ were used, including outdoor time a week, average sleep duration, MSFsc (corrected mid-sleep phase on free days), and Social Jetlag (different between mid-sleep phase on free days and work days) (67, 68). The Pittsburgh Sleep Quality Index (PSQI) were applied for general sleep quality (69). We also applied Insomnia Severity Index (ISI) to evaluate the degree of insomnia and divided subjects into two categories with cutoff 8 (ISI < 8, without insomnia; ISI ≥ 8, with insomnia) (70, 71). All of these questionnaires were collected by trained interviewers.

### Image acquisition

The DTI data were acquired on a 3-Tesla MRI system (General Electric Medical Solutions) at MR Research Center of the Xijing Hospital of the Fourth Military Medical University, Xi’an, in China. Subjects’ heads were positioned carefully with restraining foam pads to reduce head motion, and ear plugs were used to reduce the scanner noise discomfort. Prior to the DTI acquisition, high-resolution T1- and T2-weighted images were acquired for each subject by two expert radiologists to exclude the possibility of clinically silent lesions. Diffusion-weighted sequences with single-shot echo planar imaging in alignment with the anterior-posterior commissural plane were acquired with the following parameters: field of view = 240 × 240 mm^2^, repetition time (TR)/echo time (TE) = 10,000/82.4 ms, matrix = 256 × 256, slice thickness = 2 mm, and 70 continuous axial slices with no gap. The diffusion sensitizing gradients were applied along 64 non-parallel directions (*b* = 1,000 s/mm^2^) and one without diffusion weighting (*b* = 0).

### Image data processing and analyses

All DTI analysis was performed using analysis tools from FSL (FMRIB Software Library).^1^ The DTI data were corrected for eddy distortions and motion artifacts via affine registration on the first no-diffusion weighted volume of each subject by means of the FDT v2.0 (FMRIB’s Diffusion Toolbox) (72), part of FSL. The FA, MD, axial diffusivity (AD) and RD images was created by fitting the diffusion tensor to raw diffusion data after brain extraction using Brain Extraction Tool (73). Then, voxel-wise statistical analyses of the FA data were carried out using the TBSS v1.2 of FSL (74). Briefly, the FA images from all participants were non-linearly warped to the FMRIB58_FA template by FNIRT (75). Next, the mean FA image was created and thinned to create a mean FA skeleton (threshold of 0.2) representing the centers of all tracts common to the group. Each subject’s aligned FA data was then projected back onto this skeleton, and the resulting data were fed into voxel-wise permutation-based cross-subject statistics. The MD, AD and RD were performed the same analysis and projected onto the above mean FA skeleton.

### Statistical analysis

For the age, scan time, body mass index (BMI), four index of MCTQ, PSQI, insomnia and BDI score, permutation-test-based Student’s two sample *t*-tests with 1,00,000 random sampling were carried out between the AG group and AA group. For the gender, insomnia state and depressive state, χ^2^ tests were performed between the two groups.

Then, voxel-wise DTI statistics analyses were performed for FA using FSL’s permutation-based non-parametric testing (Randomise v2.1, 10,000 permutations) with a general linear model. We applied a ‘‘two groups with continuous covariate interaction’’ model^2^ to explore the difference of the linear relationship between the FA and BDI values between the two groups. The design matrix included two genotypes (AG = 1 and AA = 0), two genotypes multiply by BDI value interaction (the interaction effects), as well as columns of sex, age, body mass index (BMI) and six circadian/sleep related parameters (outdoor time a week, average sleep duration, MSFsc, Social Jetlag, PSQI and ISI index) as covariates. Previous studies had found altered apparent diffusion coefficient of water during sleep (76), and diurnal fluctuations in white matter microstructural characteristics (77) and in brain volume (78). Therefore, in order to control the effect of time of day on our results, we also added the scan time of MRI as covariates. The MD, AD and RD underwent the similar analysis for the interaction effects genotypes × BDI. Furthermore, we compared the FA between the two PER1 genotypes with the above ten parameters as covariates. Multiple comparisons across voxels were corrected using the TFCE (threshold-free cluster enhancement) method (*p* < 0.05) (79). The JHU ICBM-DTI-81 white-matter label atlas (80) was used to label significant tracts.

For the convenience of showing results, we extracted and averaged the adjusted BDI, FA, MD and RD among the voxels in each WM tract which showed significant interaction effects of genotypes × BDI. The adjusted BDI, FA, MD and RD were the measures after removing the above ten covariates. Then, we performed Pearson correlation analysis between the adjusted WM characteristics and BDI for the two groups, respectively. Significant correlation was identified when *p* < 0.05, significant correlation trend was recognized when the *p* value was between 0.05 and 0.1.

## Results

### Demographic, behavioral, and genotype characteristics

The demographic characteristics of the participants according to the PER1 genotype were presented in Table 1. The distribution of PER1 genotype in the sample was: A/A 52 (67.53%), A/G 25 (32.47%), G/G 0, consistent with the Hardy-Weinberg Equilibrium (*χ^2^* = 0.941, *p* = 0.332). Allelic frequencies were A = 83.77%, and G = 16.23%. Age, gender, BMI and scan time did not reach statistical significance between these two groups.

**TABLE 1:** Demographic characteristics and circadian rhythms associated phenotypes according to the polymorphism of *PER1* gene (rs7221412).

Variables	Total (*n* = 77)	AA (*n* = 52)	G carriers (*n* = 25)	*T* value or χ^2^ value	*P* value
Gender (*number, male/female*)^+^	50/27	34/18	16/9	0.014	0.91
Age (*year, mean* ± SD)*	20.94 ± 2.09	20.96 ± 2.07	20.88 ± 2.19	0.16	0.93
Scan time (*time, mean* ± SD)*	14.87 ± 2.90	14.71 ± 2.81	15.19 ± 3.10	–0.67	0.50
BMI (*mean* ± SD)*	21.70 ± 2.79	21.90 ± 3.07	21.29 ± 2.11	0.90	0.37
Outdoor time (*h, mean* ± SD)*	13.20 ± 9.67	13.97 ± 10.91	11.61 ± 6.30	1.00	0.33
Sleep duration (*h, mean* ± SD)*	7.65 ± 0.61	7.66 ± 0.68	7.63 ± 0.43	0.18	0.86
Social Jetlag (*h, mean* ± SD)*	0.48 ± 0.53	0.50 ± 0.56	0.42 ± 0.48	0.67	0.51
MSF_*SC*_ (*time, mean* ± SD)*	4.10 ± 0.70	4.18 ± 0.70	3.95 ± 0.67	1.36	0.18
PSQI (*mean* ± SD)*	4.36 ± 2.62	4.44 ± 2.47	4.20 ± 2.96	0.38	0.75
ISI (*mean* ± SD)*	5.01 ± 3.06	5.12 ± 3.01	4.8 ± 3.2	0.42	0.71
Insomnia state (number, with/without)^+^	15/62	11/41	4/21	0.29	0.59
BDI value (*mean* ± SD)*	7.12 ± 5.38	6.48 ± 4.17	8.44 ± 7.22	–1.51	0.14
Depressive state (number, with/without)^+^	8/69	2/50	6/19	7.37	0.007

BMI, body mass index; MSF_*SC*_, mid-sleep phase on free days corrected for the sleep deficit accumulated during the work week; PSQI, Pittsburgh Sleep Quality Index; ISI, Insomnia Severity Index; BDI, Beck Depression Inventory; SD, standard deviation. Two insomnia states were defined with cutoff 8 (ISI < 8, without insomnia; ISI ≥ 8, with insomnia). Two depressive states were identified using cutoff BDI, 14 (BDI < 14, without depression; BDI ≥ 14, with depression). *Permutation-test-based Student’s two sample *t*-test. ^+^χ^2^ test.

Behavioral data measured by several scales were compared. The feature of circadian rhythm indicated by outdoor time, sleep duration, MSF_*SC*_ and social jetlag, did not exhibit significant differences between AA and AG groups (*p* = 0.33, 0.86, 0.18, and 0.51, respectively, Table 1). Furthermore, the general sleep quality indicated by the score of PSQI, insomnia evaluated by ISI, and BDI values in the AA group did not markedly different from that in the AG group (*p* = 0.75, 0.71, and 0.14, respectively, Table 1).

11 subjects (11/52 = 21.15%) were in the insomnia state in the AG group, and 4 subjects (4/25 = 16%) in the AA group with the ISI threshold value of 8. The insomnia state did not show significant different between these two groups (*p* = 0.59). However, according to the BDI value with cutoff of 14, 6 subjects (6/25 = 24%) were in the depressive state in the AG group (25/77 ≈ 1/3), and 2 subjects (2/52 = 3.85%) in the AA group (52/77 ≈ 2/3). The AG group exhibited higher proportion of the with-depressive state than that of the AA group (*χ^2^* = 7.37, *p* = 0.007) based on the χ^2^ test. In other words, 24% (6/25) subjects with the PER1 heterozygote were in depression state among the 1/3 population and 6/8 of people with depression were the AG genotype. However, only 4% (2/52) of subjects were in depression state among the PER1 A homozygote population and 2/8 of people with depression were in the AA group.

### The alterations of fractional anisotropy between PER1 genotypes

**FIGURE 1:**
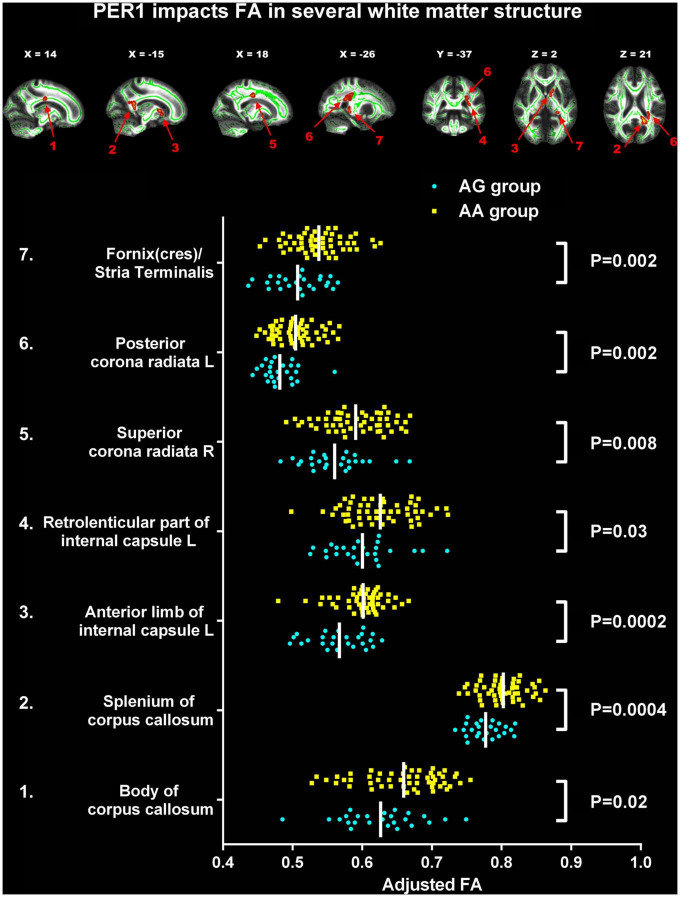
Significant changes of fractional anisotropy (FA) between two PER1 genotypes. The significant difference was observed in the following seven white matter tracts between G carrier group and AA group: 1. Body of corpus callosum (CC); 2. Splenium of CC; 3. The left anterior limb of internal capsule; 4. The left retrolenticular part of internal capsule; 5. The right superior corona radiata; 6. The left posterior corona radiata; 7. Left fornix(cres)/strial terminalis. FA, fractional anisotropy; L, left; R, right. The yellow box represents AA group; the blue dot represents the AG group.

Compared to AA homozygotes group, the AG group showed decreased FA in several WM tracts with TFCE correction (*p* < 0.05), including body of corpus callosum (CC), splenium of CC, left anterior limb of internal capsule (ALIC), left retrolenticular part of internal capsule (RLIC), right superior corona radiata (SCR), left posterior corona radiata (PCR), and left fornix(cres)/strial terminalis (Table 2 and Figure 1).

**TABLE 2:** White matter regions where *Per1* Gene (rs7221412) variants between A/A and G carriers significantly differ in fractional anisotropy.

White matter tracts	Voxels		Peaks MNI coordinates (x,y,z)		Patterns
Body of corpus callosum	40	18	–18	35	G carriers < AA
Splenium of corpus callosum	265	–18	–51	21	G carriers < AA
L anterior limb of internal capsule	125	–15	9	4	G carriers < AA
L retrolenticular part of internal capsule	22	–31	38	14	G carriers < AA
R superior corona radiata	47	19	–18	37	G carriers < AA
L posterior corona radiata	176	–26	–42	22	G carriers < AA
L fornix(cres)/strial terminalis	76	–23	–34	–1	G carriers < AA

### The interaction effect of PER1 genotype on the association between white matter structure and Beck Depression Inventory values

**FIGURE 2:**
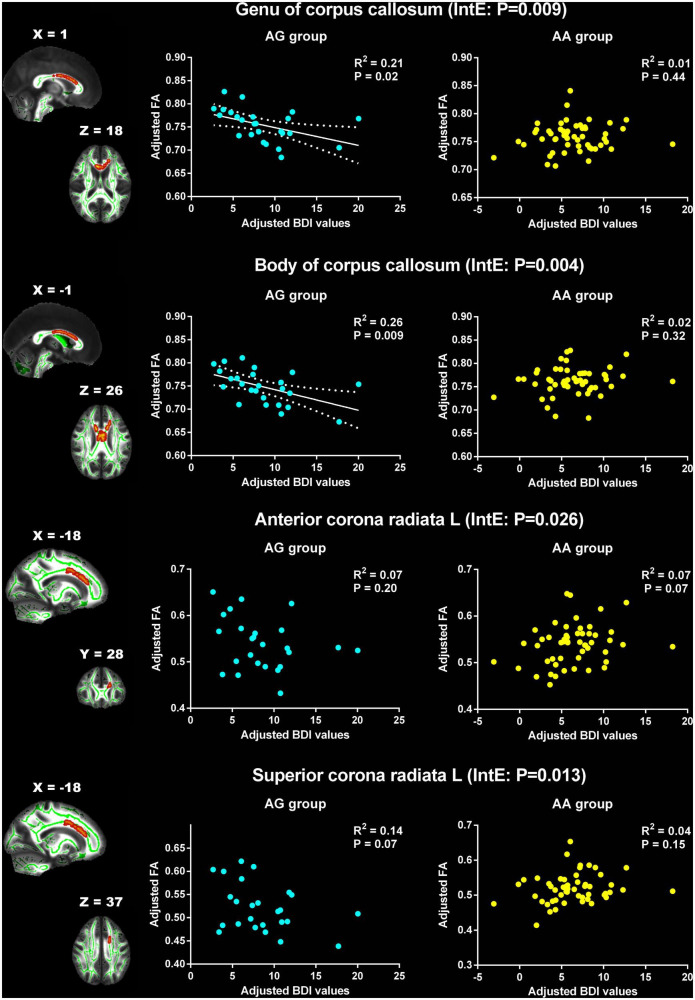
Significant effects of PER1 genotypes on the associations between BDI values and FA. The significant interaction effects of genotypes × BDI were observed in the white matter tracts of genu of corpus callosum, body of corpus callosum, the left anterior corona radiata and the left superior corona radiata. The adjusted BDI and FA were the measures after removing the ten covariates. For the convenience of showing interaction effect, we averaged the adjusted BDI and FA among the voxels in the above WM tracts, and performed the correlation analysis between the adjusted BDI value and FA of WM tracts in each group. BDI the Beck Depression Inventory; FA fractional anisotropy; L left. The blue dot represents the AG group; the yellow dot represents the AA group. InE interaction effects. The white solid line represents the linear regression of the correlation.

The associations between BDI values and FA, AD, MD and RD were explored in the two PER1 genotype groups and the significant interaction effects between genotype and BDI were present in Table 3. Significant genotype × BDI interaction effects on FA were observed in the genu and body of CC, left anterior corona radiata (ACR), and left SCR. In detail, significant negative correlation was found between the FA in genu of CC and the BDI in AG group (*R*^2^ = 0.21, *p* = 0.02), however, this correlation was not found in AA group (*R*^2^ = 0.01, *p* = 0.44). Similar results were found in body of CC. The FA in the left ACR was not related with BDI in AG group (*R*^2^ = 0.07, *p* = 0.20), but showed positive correlated trend in AA group (*R*^2^ = 0.07, *p* = 0.07). Conversely, negative correlated trend between the FA in left SCR and BDI was found in AG group (*R*^2^ = 0.14, *p* = 0.07), no relationship between them in AA group (*R*^2^ = 0.04, *p* = 0.15, Figure 2).

**TABLE 3:** Significant interaction effects between *Per1* gene variants and BDI values in white matter microstructure.

White matter tracts	Voxels	Peaks MNI coordinates (x, y, z)			Interaction effect (*p* value)	Patterns (G carriers group vs AA group)
**Fractional anisotropy**						
Genu of corpus callosum	111	5	20	16	0.009	SNC vs N.S.
Body of corpus callosum	768	5	18	16	0.004	SNC vs N.S.
L anterior corona radiata	130	–16	16	30	0.026	N.S. vs PCT
L superior corona radiata	70	–17	4	38	0.013	NCT vs N.S.
**Radial diffusivity**						
Genu of corpus callosum	29	5	20	16	0.0003	SPC vs N.S.
Body of corpus callosum	159	14	13	27	0.0001	SPC vs N.S.
L anterior limb of internal capsule	49	–13	4	6	0.01	SPC vs N.S.
L posterior limb of internal capsule	257	–23	–13	7	0.008	SPC vs N.S.
L external capsule	30	–30	–17	12	0.004	SPC vs N.S.
**Mean diffusivity**						
L cerebral peduncle	57	–19	–15	–8	0.02	PCT vs NCT
L anterior limb of internal capsule	26	–19	–1	10	0.006	SPC vs N.S.
L posterior limb of internal capsule	349	–25	–13	13	0.009	SPC vs N.S.
L retrolenticular part of internal capsule	44	–25	–19	–1	0.006	SPC vs N.S.
L superior corona radiata	101	–24	–9	19	0.01	SPC vs N.S.
L sagittal stratum (include inferior longitidinal fasciculus and inferior fronto-occipital fasciculus)	39	–40	–17	–13	0.002	SPC vs N.S.
L external capsule	196	–30	–17	12	0.002	SPC vs N.S.
L fornix(cres)/strial terminalis	42	–30	–21	–10	0.009	SPC vs N.S.

SNC, significantly negative correlation; SPC, significantly positive correlation; NCT, negative correlation trend; PCT, positive correlation trend; N.S., no correlation and trend; L, left.

**FIGURE 3:**
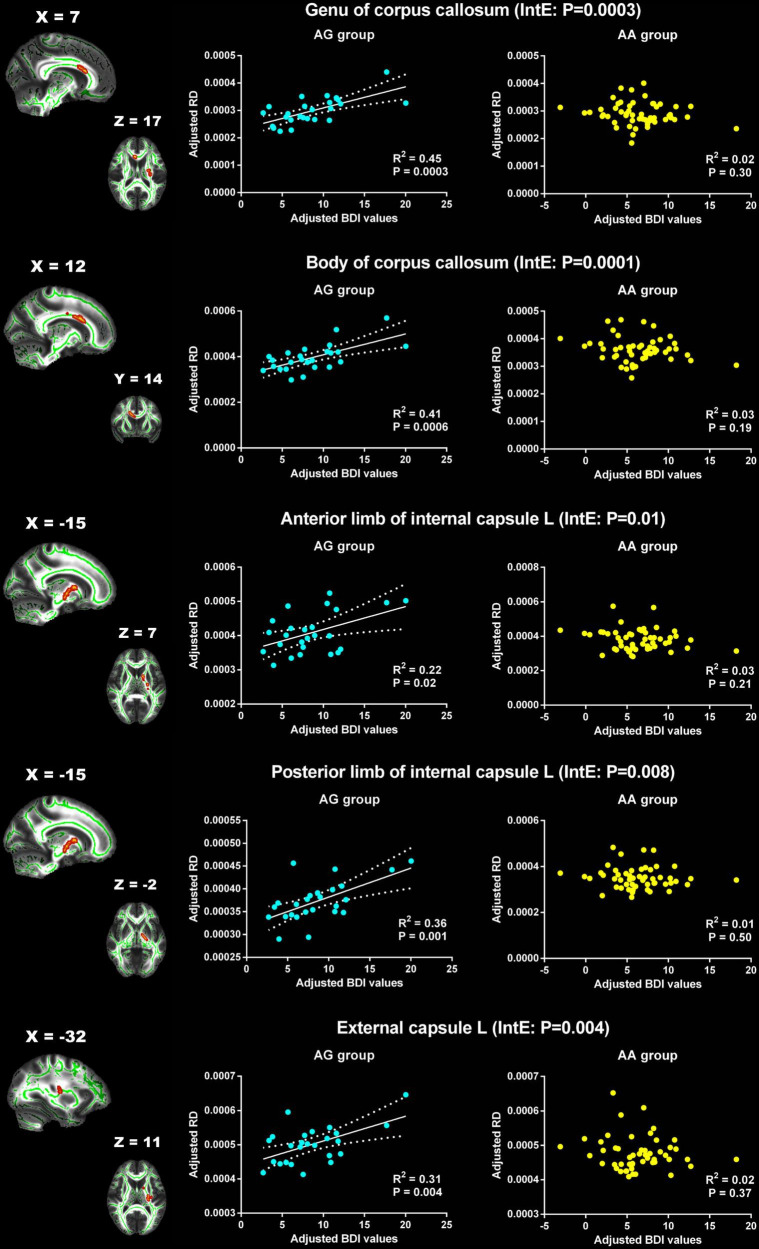
Significant effects of PER1 genotypes on the associations between BDI values and RD. The significant interaction effects of genotypes × BDI were observed in the white matter tracts of genu of corpus callosum, body of corpus callosum, the left anterior limb of internal capsule, the left posterior limb of internal capsule and the left external capsule. The adjusted BDI and RD were the measures after removing the ten covariates. For the convenience of showing interaction effect, we averaged the adjusted BDI and RD among the voxels in the above WM tracts, and performed the correlation analysis between the adjusted BDI value and RD of WM tracts in each group. BDI, the Beck Depression Inventory; RD, radial diffusivity; L, left. The blue dot represents the AG group; the yellow dot represents the AA group. InE, interaction effects. The white solid line represents the linear regression of the correlation.

For RD, the significant interaction effects were observed in genu and body of CC, left ALIC, left posterior limb of internal capsule (PLIC) and left external capsule (Table 3). The RD of these WM tracts showed significant positive correlation with BDI in AG group, but no association in AA group (Figure 3).

**FIGURE 4:**
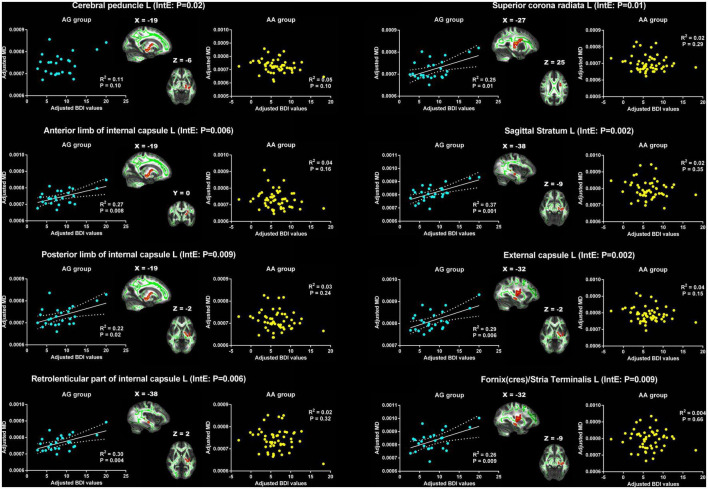
Significant effects of PER1 genotypes on the associations between BDI values and MD. The significant interaction effects of genotypes × BDI were observed in the white matter tracts of cerebral peduncle, anterior limb, posterior limb and retrolenticular part of internal capsule, superior corona radiata, sagittal stratum, external capsule and fornix/stria terminalis on the left cerebral hemisphere. The adjusted BDI and MD were the measures after removing the ten covariates. For the convenience of showing interaction effect, we averaged the adjusted BDI and MD among the voxels in the above WM tracts, and performed the correlation analysis between the adjusted BDI value and MD of WM tracts in each group. BDI, Beck Depression Inventory; MD, mean diffusivity; L, left. The blue dot represents the AG group; the yellow dot represents the AA group. InE, interaction effects. The white solid line represents the linear regression of the correlation.

For MD, the significant interaction effects were found in cerebral peduncle, ALIC, PLIC, RLIC, SCR, sagittal stratum, external capsule and fornix(cres)/strial terminalis on the left cerebral hemisphere (Table 3). The MD in the left cerebral peduncle exhibited positive correlation trend with BDI in AG group (*R*^2^ = 0.11, *p* = 0.10), but showed negative correlation trend in AA group (*R*^2^ = 0.05, *p* = 0.10). The changes of the relationship between BDI and the MD of the remaining WM tracts from the AG group to the AA group were similar. In the AG group, the BDI was significant positive correlated with MD. However, in the AA group, no significant relationship was found between them (Figure 4). However, for AD, no significant interaction effects were found.

## Discussion

In the present study, we investigated the effects of PER1 (rs7221412) gene polymorphisms on the associations between WM integrity and depression level (measured by BDI) using TBSS analysis. We found that more subjects with PER1 heterozygotes were in depression state (BDI ≥ 14) than AA group, and showed poorer WM integrity in CC, internal capsule, corona radiata and fornix. Furthermore, significant relationships were found between BDI and WM microstructure characteristics in the above WM tracts, external capsule and sagittal stratum in AG group, but not in AA group. These results suggested that the poorer WM integrity in several WM tracts may be associated with the increased depression risk especially in subjects carrying variants of PER1 gene.

In our present study, no significant difference was found in circadian rhythm, sleep quality and insomnia between AG and AA groups. Lim et al. have explored the associations between polymorphisms of PER1 and the timing of behavioral rhythms measured by actigraphy in 537 Europeans, and found that mean activity timing was delayed by 67 min in GG group versus AA group (58). 184 subjects (34%) were AA, 263 subjects (49%) were AG and 90 subjects (17%) were GG among the 537 Europeans, which suggested that the distribution of PER1 was AA:AG:GG ≈ 2:3:1 in Europeans. However, we found that 67.53% were PER1 homozygous A and 32.47% were PER1 heterozygotes among 77 subjects, but no subjects were the PER1 homozygous variant G, which suggested that the distribution of PER1 was AA:AG:GG ≈ 2:1:0 in Chinese Han population. The lower allelic frequencies of G may be one of reasons why there were no significant changes of circadian rhythm/sleep-related behavioral data between AA and AG group. Furthermore, previous studies have found that several gene polymorphism frequency varies depending on both region and ethnicity in healthy and clinical diseases populations (81–84). In addition, the genotype distribution of PER1 in the present study was identified in a small sample of 77. Therefore, further studies could explore the effect of PER1 polymorphism on these behavioral data and verify these inconsistent results whether were due to ethnicity in a large population.

One interesting observation in our present study was that the AG group exhibited higher proportion of depression state with BDI cutoff of 14 than that of the AA group. These results indicated that subjects with PER1 heterozygote showed higher depression risk and more depression symptom compared with other persons. Previous research have found that several gene variations were associated with depression vulnerability, such as PER2 (34), TOMM40 rs2075650 SNP (85), 5-HTTLPR/BDNF Val66Met (86–88), CRHR1 (89) and COMT (90). Our findings were the complement for the association of the gene polymorphism with depression risk. Furthermore, Li et al. have found blunted diurnal rhythms in the expression of PER1 in MDD patients and these disruptions persisted 8 weeks after treatment (32). Therefore, combined our results, these findings suggested that PER1 gene may play a role in depression risk. Further studies were needed to verify our results with large sample.

Another interesting observation in our present study was that besides the higher depression proportion, the AG group exhibited poorer WM microstructure integrity than the AA group, mainly in CC, internal capsule, corona radiata and fornix. These WM tracts have been reported to have decreased FA in MDD (91–94). Vulser et al. have investigated the association of subthreshold depression with WM microstructure alterations in adolescents and found that decreased FA in the anterior body and genu of CC in adolescents with subthreshold depression (95). Our findings were consistent with these researches and suggested that subjects with PER1-G heterozygote with lower FA may probably develop to the subthreshold depression or MDD. In order to explore the effect of PER1 on the relationship between WM characteristics and depression level, we analyzed the interaction effects of genotype × BDI on WM characteristics. Significant correlations were found between BDI and WM characteristics in CC, internal capsule, corona radiata, fornix, external capsule and sagittal stratum in AG group, but not in AA group. The CC plays an important role in the inter-hemisphere information communication, and is critical for emotional regulation and associated with cognitive functioning (96). The corona radiata is part of the limbic-thalamo-cortical circuitry and is composed of ascending and descending fibers that relay information to and from the cerebral cortex. Several researches have indicated that the corona radiata is implicated in emotional and executive processing, major affected functions in MDD (97, 98). Other WM tracts also have been reported involving the mood and cognitive function (99–102). These results suggested that PER1-G heterozygote, 1/3 of Chinese college students, may be more prone to depression if they exhibited lower FA and higher RD and MD in emotion-related WM tracts. However, in AA group, we did not find these relationships. Therefore, PER1 gene polymorphisms and WM microstructure may be promising indictors for early identification of depression risk.

Emerging studies have showed that more than 90% of depressed patients reported disruptions in sleep including insomnia and early morning awakening. These patients tend to have more severe forms of MDD and may be at an increased risk for suicidal ideation and suicidal behaviors (20, 103, 104), which supports a circadian hypothesis of depression that based, in part, on data showing that a subgroup of depressed patients has dysregulated 24 h rhythms including sleep, hormonal secretions, core body temperature and mood (17, 24, 105, 106). Perhaps the strongest and most direct evidence for a circadian defect in depression comes from a study of postmortem 24 h sinusoidal gene expression rhythms across six regions of human brain showing a dramatic dysregulation of circadian genes in MDD compared to controls (19). This article has further revealed that the expression of core clock genes, including Period genes (PER1-3) was different between controls and MDD patients. PER genes regulate circadian rhythm by repressing the transcriptional activity driven by upstream rhythm genes, and inhibiting their own expression through a negative autoregulatory feedback loop that cycle in about 24 h (107, 108). Besides, PER genes show a staggered phase relationship, with PRE1 peaking soon after sunrise, PER3 peaking during midday, and PER2 peaking in the afternoon (19, 109, 110) while this circadian pattern was weak in MDD patients (19). A genome-wide study had found a strong relationship between sleep deprivation and the expression of PER genes in MDD and bipolar disorder patients (111). Moreover, the role of the circadian clock in the homeostasis of stem cells and in the regulation of cellular development including differentiation across tissue subtypes is supported by numerous studies (112–114). And the circadian rhythm disorder may disrupt normal sleep rhythm, which promotes myelination and oligodendrocyte precursor cells proliferation and is associated with higher expression of genes coding for phospholipid synthesis and myelination in oligodendrocytes (115–117). These brain alterations caused by circadian clock genes have been related to neurogenesis, and would further associated with psychiatric disease including depression (118, 119).

The present study has several limitations that should be considered. First, large samples of future researches were needed to validate our present study. Second, the current study was cross-sectional but not longitudinal. Whether subjects in the AG group with lower FA and higher MD and RD in specific emotion-related WM tracts really develop to depression should be investigated in the future longitudinal studies. Third, we measured the depression state by BDI in healthy college students. Further studies should verify our results in depressed people. Forth, we found that several emotion-related WM tracts were associated with BDI in AG genotype. However, our present study did not investigate the performance of emotion-related tasks. Future research should explore the effect of PER1 polymorphism on the association of WM microstructure integrity with specific emotion-related task.

## Conclusion

In conclusion, decreased FA in several WM tracts were shown in PER1 (rs7221412) AG group compared with the AA group, and PER1 genotype had an interaction effect on the associations between the WM microstructural integrity in emotion-related WM tracts and depression level. These findings suggested that the PER1 gene polymorphisms and WM characteristics in several emotion-related regions may provide effective measures of prediction for depressive risk at an early phase. Future studies should validate these results in large sample.

## Data availability statement

The raw data supporting the conclusions of this article will be made available by the authors, without undue reservation.

## Ethics statement

The studies involving human participants were reviewed and approved by the Institutional Research Ethics Committee of the Xijing Hospital of the Fourth Military Medical University. The patients/participants provided their written informed consent to participate in this study.

## Author contributions

RZ, J-BS, HD, and X-JY were guarantors of integrity of the entire study. RZ, J-BS, L-ML, and WQ contributed to study concepts/study design. RZ, XL, F-MW, Z-YH, and M-YC contributed to data acquisition. RZ, J-BS, HD, L-ML, C-ZT, and N-GX contributed to data analysis/interpretation. RZ, J-BS, HD, and CC contributed to manuscript drafting or manuscript revision. All authors contributed to manuscript revision and read and approved the submitted version.

## Footnotes

1
http://www.fmrib.ox.ac.uk/fsl

2
https://fsl.fmrib.ox.ac.uk/fsl/fslwiki/GLM#Two_Groups_with_continuous_covariate_interaction
